# Diptheria

**Published:** 1865-04

**Authors:** James S. Whitmire

**Affiliations:** Metamora, Ill.


CHICAGO
MEDICAL JOURNAL.

VOL XXII.] APRIL, 1865. [No. 4.

### ORIGINAL COMMUNICATIONS.

### DIPHTHERIA.

By JAMES S. WHITMIRE, M.D., of Metamora, Ill.

*Editors Chicago Medical Journal:*

During the months of December, January, and to this time (Feb. 19th), in the German settlement, four miles west of this place, Diphtheria has extensively prevailed, in an unusually malignant form, and from the fact that it has been nearly exclusively confined to one township, I can view it in no other light than that of an epidemic; almost all the children in that vicinity having been affected with it in a greater or less degree.

Why it should be confined to that particular locality, and not extend its ravages over a greater extent of country, is something very incomprehensible to me. There is nothing peculiar in the topography of that locality that would seem to indicate any local influence that might be likely to produce a detoriation of the vital forces from poisonous effluvia, either from natural or artificial causes, it being high and dry, situated on the edge of the timber, and with a beautiful prairie in front, gradually descending towards the south. The timber in the rear is nearly all cleared from the surface, which is beautifully undulating, and descending to the north, forming deep ravines, where there are crystal running brooks. The fields, in front and rear, are well cultivated, and the inhabitants industrious, temperate and frugal. They are what may be called good livers generally, but probably use more pork and sour-krout as a common diet than twice their number of Americans would do. I therefore can see no appreciable cause for this terrible scourge falling upon them, excepting that of an epidemic influence, which is wholly unaccountable to me.

We have had a dry, cold winter, mercury ranging from 32° above to 10° below 0, and no rain of any amount has fallen since Nov., 1864, until the 2d and 3d of Feb., so that, all things considered, we have had one of the most even and pleasant winters that I have seen for many years.

Viewing the character of this scourge of youth, as I do, which is that of blood-poison, without stopping to enquire of what that poison consists, or the manner of its introduction into the system, whether from external influence, or whether it is engendered within, from a vitiated condition of the circulating fluid, and hence an unhealthy molecular formation of the tissues; of these things I leave for future investigations by ætiologists. My own opinion is, that the disease is engendered within, but probably more or less influenced by external influences.

Persons attacked with diphtheria, have generally complained of soreness of the pharynx, fauces, and tonsils, general languor, lassitude, and indisposition to take food for three or four days previous to the occurrence of any febrile excitement becoming apparent. At this time on examining the throat, an immense injection of the submucous cellular tissue, and œdema of the tonsils, soft palate, and uvula is present, not amounting to a sthenic grade of inflammation of these parts; and at this period patches of exudation of pseudo-membrane of a light straw color begin to appear, seldom covering the whole engorged part, and febrile action, with great quickness, and often frequency of pulse, is nearly always an accompaniment of this stage of the disease.

There is also more or less inflammation of the cellular tissue of the neck, about the angle of the jaw, and difficult articulation on account of the swelling, influenced more or less by the functional derangement of the cervico-spinal nervous system. The languor and weakness that occurs after the incipient stage is, no doubt, owing to the failure of the vital forces to produce the natural quantity of red globules in the blood, consequently it fails to produce a sufficient absorption of oxygen for the ordinary wants of the system, and hence the production of fibrin is lessened in quantity, and detoriated in quality, producing important molecular changes in the formation of the tissues, and necessarily follows, in consequence functional derangement of all the secretory and excretory organs, and as a consequence, there is a scanty elimination of excrementitious matter contained in the circulating fluid; and hence the fever in this disease is of a decidedly asthenic character, and the irritable, frequent pulse which always accompanies the worst form of this disease is, no doubt, owing partly to the irritation of the internal lining, or endangium of the heart and blood-vessels, in connection with the unhealthy nutrition and consequent functional derangement of the great nervous centres.

The idea is very general among the people that this is simply a local disease, and that if the throat is once cured by proper applications, the patient will necessarily recover; and or this erroneous opinion physicians are very much to blame, for instead of talking to the friends and treating them as in telligent beings, they make an unwarranted display of swabs, caustics, pencils, and washes; look wise, and say nothing, in order to impress the family with their superior wisdom and the great and imminent danger of their patients. This is radically wrong. On the contrary everything nécessary to be done should be explained in, as nearly as practicable, the most simple manner, and the prognosis of this disease should be given in all cases in the most cautious manner possible; for there is no stage in which there is not great danger from its assuming a malignant form, or that the tendency may not be to the extension of the specific inflammation into the larynx producing what is usually called pseudo-membranous croup. In observing these rules, and then in ordering medicines or a particular regimen, your directions will be more likely to be attended to intelligibly and with greater care, so that your patients will be more secure from the intermeddlings of officious people.

On account of this general impression (that of its being simply a local disease), the physician is seldom consulted until the exudation begins to appear, and the difficulty of articulation creates alarm in the minds of the friends. However, under these circumstances, I usually administer a moderate dose of castor oil in preference to any other laxative, as it has less tendency to debilitate the system than other medicines of this class. I also pencil the tonsils and throat with a solution of iodine; ℞. Tinct. Iodine, ʒ ij; Pot. Iod., gr. iv; Aqua, ℥ j; taking great care not to interfere with or rub off the exudation, further than the solution affects it by softening and dissolving the membrane; thus imitating nature to some extent of getting rid of it, and in this respect it possesses an influence over the ordinary remedies, caustics, etc., they coagulating and hardening the exudation. Besides, the iodine acts as a local capillary tonic whenever it comes in contact with the surfaces, and enables the engorged vessels to relieve themselves of their contents, and by absorption to get rid of the œdema consequent upon the engorgement.

For the constitutional remedies I prescribe, Pot. Chloras.,

ʒ iv; Ammon. Mur., ʒ ij; Sp'ts Nit., ʒ ij; Creosote, gtt. viij; Tinct. Ferri Mur., ʒ ij; Aqua, ℥ iijss. Dose. One dessertspoonful in sweetened water, every four hours; this is for a child from five to eight years old. If there is great debility, I give Pulv. Dov. and Quinine. My reasons for using this mixture are plain, simple, and easily adduced, and it is eminently calculated to fill all the indications of treatment manifested in the character of the disease.

The chlorates by their solution in the blood, facilitate the absorption of oxygen; the chalybeate replenishes the diminishing red corpuscles, and, together rendering the capacity of the blood greater for the reception of Oxygen and thus rendering its more healthy constitution available in secretion, absorption and in the formation of the tissues; the creosote is a direct antiseptic, and thus with the revivification of the blood, by the absorption of oxygen, they must necessarily play an important part in the formation of more healthy tissues, and in connection with this, the great nervous centres are relieved from the depressing influence of vitiated blood, thus allowing its natural influence over the secretory and excretory functions, and establishing an equilibrium of the circulating fluid, by means of the restoration of this great central influence. The influence, also, of diet, in the treatment of this disease, is by no means to be overlooked. And it matters very little what method of treatment be adopted unless the patients are well-nourished from the beginning, the chances are that there will be a great mortality among them; hence I give a decided preference to rich animal broths, frequently given. Milk and a little cooked fruit, or roast potatoes, three times a day, to give the stimulus of distension. The stomach and vital forces being in no condition to assimilate solid food, is the reason for not allowing it any oftener.

I seldom or never pencil the fauces and tonsils with the solution of iodine more than three or four times, once a day. Instead of this I prepare a gargle, which I use as such, or pencil the tonsils or affected part, as the case may be, with the same, four or five times a day, or oftener, if the throat is very painful. This gargle is composed of ℞. Alum Sulph., ʒj; Alcohol, ℥j; Acid Aromat. Sulph., ʒj; Creosote, Oil Cinnam., āā, gtt. xl; Treacle, ℥iij. This mixture is very stimulating to the parts, and children do not like to use it, but it has a soothing influence on the tender surface, and aids the patient materially in clearing the throat of the tenacious mucus that accumulates on the diseased surface; aside from this may not the vapor of the creosote and oil that is necessarily inhaled, exert a beneficial influence from its local stimulation of the upper air passages, in preventing the exudation of the pseudo-membrane in the larynx and trachea, which not unfrequently occurs?

So far as my limited observation goes, I am decidedly of the opinion that in this respect the influence thus exerted should not be lightly regarded, and for the purpose of availing myself of whatever beneficial influence they do exert, I direct my patients to inhale them by means of a solution in warm water, put in a wide-mouthed bottle, well-corked, and two tubes through the cork, one going nearly to the bottom of the bottle, and the one for the mouth-piece simply going through the cork. It is a cheap and simple apparatus that can be prepared with very little ingenuity in almost every house. Under this course of treatment as a standard, during this epidemic, there has been forty well-marked cases of which I have had charge. Of the whole, I have lost two cases, one being 18 months, the other 10 months old. This is a ratio of 5 per ct. But if, like many other of my professional brethren are prone to do, I should call every slight affection of the throat that I am called upon to see or prescribe for, diphtheria, then I would not lose the half of one per cent., and though very many of such cases are evidently a mild character of diphtheria, yet it is unwise to make a great "ado about nothing," and alarm the friends without benefiting the patient in the least.

Now, to conclude this already too extended article, I trust that it will not be considered inappropriate, or ostentatious in me to give, in a concise manner, the history of a single case that occurred in the same neighborhood during the prevalence of this epidemic.

Mary Gingrich, 6 yrs. old, daughter of Christian Gingrich, whose other children, seven in number, had all had the disease, was attacked about the middle of Dec. last with diphtheria. Under the general treatment, as hereinbefore detailed, she slowly recovered, so that for two weeks she had ceased to take medicine, and played about the house.

On the 17th of January, she became so hoarse that she could not speak above a whisper, and seemed to have no other serious indisposition, so that her parents supposed it to be only a cold from which she would soon recover.

On the night of the 18th, her breathing became so difficult that her parents were much alarmed, and the child was almost uncontrollable on account of impending suffocation. As soon as daylight on the morning of the 19th they sent for me. I arrived at 10 o'clock, A. M., and found her breathing with great difficulty, the air seeming to pass through a tube not larger than a goose-quill.

On account of the prevalence of diphtheria in the neighborhood, and in the family in particular, and her recent recovery from that disease, I diagnosed at once pseudo-membranous or diphtheritic croup. I explained to the parents the imminent danger of speedy dissolution, and that there were no means of saving the child's life but to open the trachea, and even that I did not propose as a radical cure, but merely as a means that might be used for the purpose of gaining time for medication, and that the opening would render the local application of remedies more eligible.

Seeing that death was inevitable in a very few hours at most, they consented to the operation, and I, assisted by my brother and partner, Dr. J. H. Whitmire, and our students, Ray and Lamson, proceeded to open the trachea immediately below the cricoid cartilege, dividing three of the rings. There was considerable hemorrhage during the operation, both venous and arterial, the latter no doubt, coming from wounding the isthmus of the thyroid gland. We took the precaution, however, to stop the bleeding by the use of the ligature, before opening the trachea.

In order to separate the opposite sides of the wound, to admit the air more freely, we had recourse to the use of a curved needle made for the purpose, with which we took a stitch on each side, vertically under the middle ring, and fastened the thread behind the neck, which answered every purpose of free respiration. After she had become quiet, I injected the larynx from the opening, with the solution of Iodine of the same strength as before mentioned for penciling, and in doing this I used one of Dr. Warren's shower syringes which is an admirable instrument for this purpose.

On the following day, 20th, I again injected the larynx with the same solution, reduced with water one-half. On the 21st I repeated the injection, still more reduced in strength, and on account of a severe cough having set in, and a tenacious glary mucus from the bronchii, almost threatening suffocation, I determined to inject them also with the weak solution last used.

On the 22d I found her cough very much relieved, and expectoration less profuse. I now removed the threads and allowed the edges of the trachea to approximate more closely, when I perceived that she breathed more freely through the mouth. After this I used no more injections.

From the first I put her upon Mur. Tinct. Iron, Creosote, Spirits Nitre, Chlor. Pot. and Mur. Ammon. in water, with a few drops of fluid Ext. Hyosciamus. During the day Quinine in tonic doses with Dover's Powders. I enjoined the free use of chicken or beef tea as a common drink.

And now, four weeks after the operation, my little patient sits up every day, eats and drinks whatever she wants, and the wound in the neck is very near closed, so that no air escapes from it, except when she coughs, and I now consider her entirely out of danger. Now, whether my patient would have recovered so speedily, or at all, without the use of the Iodine injections, of course must remain forever unknown, but certainly there has no apparent evil results followed its use, and I am inclined to give it a decided preference over other applications, on account of its softening or dissolving power over the exudation, as well as its peculiar action upon the engorged capillaries of the part.

